# Patient-Derived Xenograft Models of Pancreatic Cancer: Overview and Comparison with Other Types of Models

**DOI:** 10.3390/cancers12051327

**Published:** 2020-05-22

**Authors:** Patrick L. Garcia, Aubrey L. Miller, Karina J. Yoon

**Affiliations:** Department of Pharmacology and Toxicology, University of Alabama at Birmingham, Birmingham, AL 35294, USA; plgarcia@uab.edu (P.L.G.); aubrey44@uab.edu (A.L.M.)

**Keywords:** patient-derived xenograft, pancreatic cancer, preclinical study, genetically engineered mouse models, 3D organoids, preclinical drug evaluation, precision medicine

## Abstract

Pancreatic cancer (PC) is anticipated to be second only to lung cancer as the leading cause of cancer-related deaths in the United States by 2030. Surgery remains the only potentially curative treatment for patients with pancreatic ductal adenocarcinoma (PDAC), the most common form of PC. Multiple recent preclinical studies focus on identifying effective treatments for PDAC, but the models available for these studies often fail to reproduce the heterogeneity of this tumor type. Data generated with such models are of unknown clinical relevance. Patient-derived xenograft (PDX) models offer several advantages over human cell line-based in vitro and in vivo models and models of non-human origin. PDX models retain genetic characteristics of the human tumor specimens from which they were derived, have intact stromal components, and are more predictive of patient response than traditional models. This review briefly describes the advantages and disadvantages of 2D cultures, organoids and genetically engineered mouse (GEM) models of PDAC, and focuses on the applications, characteristics, advantages, limitations, and the future potential of PDX models for improving the management of PDAC.

## 1. Introduction

Patients with pancreatic cancer (PC) have a uniformly poor prognosis. It is estimated that 57,600 men and women in the US will be diagnosed with PC and that 47,050 patients will die from this disease in 2020 [1]. Currently, PC is the fourth leading cause of cancer-related deaths in the US, with an overall 5-year survival rate of 9%, among the lowest survival rate for all solid tumors [1]. PC is projected to become second only to lung cancer as the leading cause of cancer related deaths in the US by 2030 [2]. Established risk factors include increasing age, tobacco smoking, type II diabetes, and obesity [3,4,5,6]. The most common form of PC is pancreatic ductal adenocarcinoma (PDAC). This subtype of PC derives from the ductal cells of the exocrine pancreas and accounts for >90% of all PC cases. Activating point mutations of *KRAS* occur in 95% of cases, and inactivating mutations or deletions of *CDKN2A* (90%), *TP53* (75%), and *SMAD4* (50%) frequently appear early in the course of disease [7,8,9]. In 2008, the first comprehensive study found that any one pancreatic tumor contained an average of 63 genetic alterations affecting 12 core cellular signaling pathways indicating the genetic heterogeneity of this disease [10]. A follow up study recently published, examined 150 pancreatic tumors using an integrated multi-platform approach examining genomic, transcriptomic, and proteomic profiles of each tumor [11]. The study found that excluding the high prevalence of *KRAS* mutations in PC, 42% of patients had at least one other alteration in their tumors with a drugable target. Therefore, those patients whose tumors harbored those alterations would be eligible for the trial designed to target that specific mutation. This study suggested a utility of patient-derived xenograft (PDX) models in personalized approaches to the treatment of PC [11]. 

About 80% of PDAC patients present with advanced stage disease, due to the paucity of specific symptoms, established biomarkers, and lack of early diagnostic methods available in the clinic. Approximately 20% of patients present with disease amenable to surgical resection, the only curative option in PDAC [12]. Gemcitabine, initially approved in 1996, has been frontline treatment for PC based on data demonstrating that it improved median survival from 4.41 months with 5-fluorouracil to 5.65 months, and also increased 1-year survival from 2% to 18% [13]. More recently, the approval of FOLFIRINOX (leucovorin, fluorouracil, irinotecan, and oxaliplatin) and the combination of gemcitabine plus albumin bound paclitaxel (nab-paclitaxel) have been approved for the treatment of advanced pancreatic cancer [14,15]. FOLFIRINOX improved median survival for patients with metastatic pancreatic cancer from 6.8 months with gemcitabine alone to 11.1 months [14]. Additionally, the combination of gemcitabine plus nab-paclitaxel increased overall survival to 8.5 months, compared to 5.7 months with gemcitabine alone [15]. This combination also improved 1-year survival [15]. Despite some improvement in median survival by these combinations, virtually all patients diagnosed with nonresectable PC die from their disease. 

**Figure 1:**
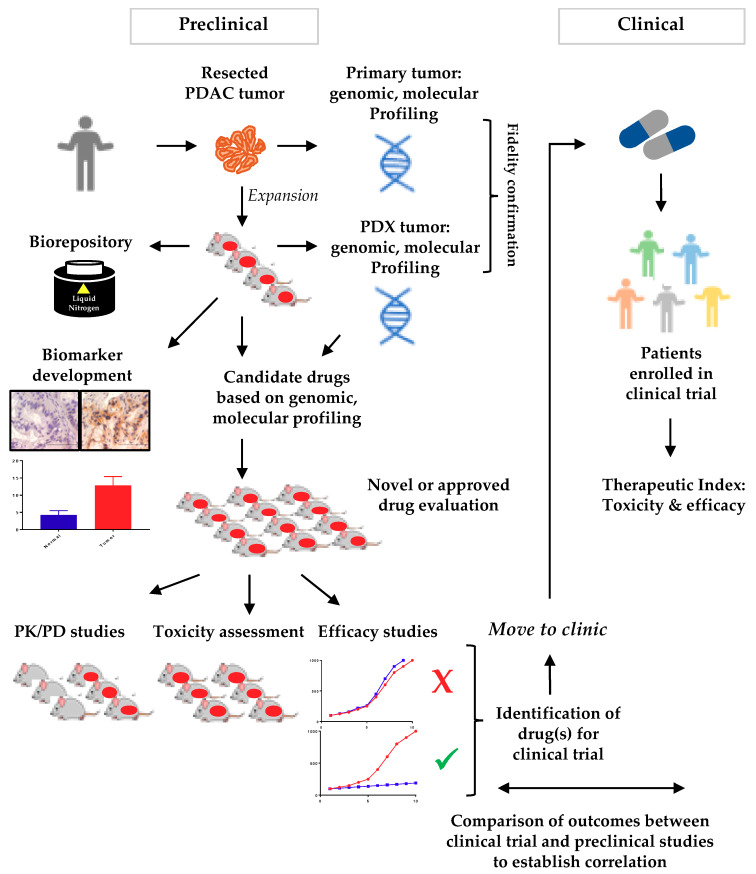
The utility of patient-derived xenograft (PDX) models in pancreatic cancer research toward precision medicine. A portion of surgically resected tumors from patients is directly implanted into immunocompromised mice and expanded for additional applications. The expanded tumor tissue can be cryopreserved for future use and analyzed for model fidelity. Panels of pancreatic ductal adenocarcinoma (PDAC) PDX models can be used in biomarker development or novel drug evaluation studies. Promising candidates can be moved into clinical trials for evaluation and a direct comparison can be made between patients and PDX models to identify the best therapeutic option for patients. Abbreviations. PK: pharmacokinetic, PD: pharmacodynamic.

Preclinical models of PDAC are essential to improving our understanding of genetic and molecular etiologies of this disease, and for developing and validating effective treatments. A variety of models have been reported. These models include immortalized cell lines (2D cell culture), 3-dimentional (3D) organoids culture system, and genetically engineered mouse models (GEMMs). A fourth model system, and the focus of this review, is patient-derived xenografts (PDXs), which are generated by direct implantation of human tumor tissue into immunocompromised mice. The goal of this review is to summarize literature characterizing the four types of PDAC models, and to discuss advantages, limitations, and potential uses of each type of model with a particular focus on PDX models of PDAC. The following section (Section 2) will discuss major model systems used in PDAC, including 2D cell culture, 3D culture (organoids), and GEM models. The rest of the review (Section 3) will be an in depth discussion of the importance of PDX models and their utility in PDAC. Figure 1 summarizes the utility of PDX models in PC research to identify agents with an ultimate goal to improve patient outcome.

## 2. Alternative Models to PDXs in PDAC

### 2.1. 2D Culture: Cell Lines and Primary Cultures

A major advancement in the development of preclinical models was the ability to propagate immortalized human cancer cells in vitro. The first pancreatic cells derived from tumor tissue and maintained in an in vitro setting were described in 1963 [16]. A second pancreatic cancer cell line, Panc-1, has been in continuous use since it was reported in 1975. The Panc-1 cell line was derived from poorly differentiated primary tumor tissue, of a 56 year old male patient [17]. Since that time, over 20 human PC cell lines have been established from primary tumor tissue. Deer et al. has reviewed the most commonly used pancreatic cancer cell lines, with respect to origin, genotype, phenotype, and tumorigenic properties [18]. 

The major advantages of PC cell lines propagated as monolayer cultures are that these cell lines are cost effective, easy to maintain and manipulate, relatively homogeneous, are subject to fewer regulatory issues than primary tumor tissue, and are available in sufficient numbers to confirm reproducibility of data produced in vitro or to establish subcutaneous xenografts in murine hosts. While a variety of concepts can be evaluated, PC cell lines are particularly useful for an experimental design that rely on using models that have 100% of cells express a transfected construct or harbor a given mutation. PC cell lines also have specific limitations. 

Firstly, pancreatic cancer cell lines do not always retain key characteristics of their tumors of origin. It is likely that clonal drift and/or expansion, adaptation to growth as monolayers on plastic, and genetic changes associated with immortalization occur with time in culture. Secondly, cell lines lack supporting stromal tissue. Primary PDAC tumors, for example, are characterized by dense fibrotic stroma that contributes to tumor progression [19,20,21]. An alternative to conventional cell line cultures is the establishment of cell lines from primary pancreatic tumor cells. Two methods used to generate this type of model involve mechanical isolation or separation of pancreatic tumor cells based on their relatively greater motility compared to most nonmalignant cells, and the subsequent use of early passage generations of the resulting cell populations [22,23,24,25]. These early passage PC cell populations are more heterogeneous and therefore more likely to mimic characteristics of primary pancreatic tumor cell populations than cells cultured long-term [26]. These early passage pancreatic primary culture cells may contain tumor-associated fibroblasts, but usually do not retain other stromal components with serial passage. However, the availability of large numbers of early passage cells may limit the types of studies that can be performed. 

### 2.2. 3D Culture: Organoids

Organoids are 3D structures derived from embryonic stem cells (ESCs), induced pluripotent stem cells (iPSCs), somatic stem cells, or tumor cells [27,28,29,30]. A primary characteristic of 3D culture is to prevent cells from attaching to the bottom of the culture dish by keeping them in suspension through the use of a matrix structure, such as collagen or Matrigel. This model system is thought to bridge the gap between 2D culture and in vivo models, as organoids can be co-cultured to contain multiple cell types such as stromal components similar to the in vivo counterpart [31,32]. Organoids derived from stem cells have been observed to differentiate and organize into structures that mimic the structure and function of the organ from which the stem cells were obtained [33,34,35].

Initial studies using this type of model focused on culturing normal pancreatic tissue [36]. These studies demonstrated that human pancreas cells embedded in Matrigel or rat tail collagen proliferated, expressed pancreatic ductal cell markers, and formed structures similar to endocrine islets [37,38]. Early approaches did not include in vitro passage and propagation of organoids, but were important to understanding pancreatic development and function [39,40]. More recently, a collaborative effort by the Tuveson and Clevers groups generated 3D cultures of murine and human PDAC cells, and established methods for serial culture [41]. Methods published in this study allowed for the culture of normal pancreas and PDAC under the same conditions [41]. Of note, when PDAC organoids were used to establish orthotopic tumors in mice, these investigators observed progression from pancreatic intraepithelial neoplasia (PanIN) precursor lesions to invasive adenocarcinoma [41,42]. Published methods from these groups describe conditions for propagation and cryopreservation, suggesting the utility of the method for proteomic, biochemical, and transcriptional analyses [41,42,43].

Organoids developed from pancreatic tumors have both advantages and disadvantages compared to 2D models. Advantages are the following. First, tumor cells grow suspended in a matrix that mimics a basement membrane and more of an in vivo setting. Second, pancreatic tumor cells can be co-cultured with stromal components to better understand cell to cell interactions in these compartments. Third, a given ‘organoid system’ can be established from a relatively small primary tumor specimen. Fourth, similar to 2D models, this model system is amenable to a wide variety of biochemical assays, as well as, drug efficacy studies. The disadvantages of this model are the following. First, this model system is still artificial and the genetic selection for growth in an artificial environment is not completely understood [44]. Second, these models cannot fully replicate the tumor microenvironment seen in human disease and lack a vasculature component [45,46,47,48]. Third, these models cannot sufficiently address the immune component in human PDAC [45,49,50]. To overcome this disadvantage, several laboratories co-culture PC cells or organoids developed from resected tissue with cancer associated fibroblasts (CAFs) in a Matrigel extracellular matrix overlayed with media containing T-cells or monocytes. The co-culture models are being used to study the crosstalk between pancreatic tumor cells, CAFs, and the immune cells by assessing the migration and invasion of T-cells into extracellular matrix, analyzing the cytokine profile of the co-culture, and assessing changes in monocyte phenotype. These models may also provide a system for evaluating the effectiveness of immunotherapies for the treatment of PDAC in vitro [31,51]. Despite these drawbacks, pancreatic organoid models can help bridge the gap between conventional cell lines and in vivo studies.

### 2.3. Genetically Engineered Mouse Models (GEMMs)

As mentioned previously in this review, a key issue in modeling PDAC is the ability to faithfully recapitulate disease. The Tuveson laboratory provided a major breakthrough in the area of pancreatic tumors, when they used a Cre/LoxP strategy to express a mutant KRAS allele specifically in pancreatic progenitor cells, resulting in the development of PanIN lesions [52]. PanIN lesions progressed to invasive and metastatic disease. The principle on which the model is based is the mutant Lox-Stop-Lox (LSL)-KRAS^G12D^ knock-in allele, which is silenced by a loxP- flanked stop element and is activated by Cre recombinase, thus activating the mutant KRAS allele. This model mimics characteristics of human disease in that tumors are associated with a strong desmoplastic stromal reaction [52,53,54]. This was the first in vivo model of pancreatic cancer that progressed from precursor lesions to metastatic disease. Several strains of mice have been used to target the pancreatic progenitor cells and include the PDX1-Cre and Ptf1a^+/Cre^ strains. A consequence of this system is that the KRAS^G12D^ mutant allele is activated in embryonic pancreatic development, which does not mimic what is observed in sporadic PDAC development. In addition, the targeting of pancreatic progenitor cells is not completely specific. For example, PDX1 is expressed in the embryonic foregut (stomach and duodenum) [55]. To address these issues, several laboratories developed tetracycline-inducible expression of mutant KRAS^G12D^ allele in pancreatic tissues, and administration of doxycycline initiates KRAS^G12D^ tumorigenesis that develop into PDAC [56,57]. Because of this, the potential of off-target tumorigenesis should be considered when designing experiments.

Another issue with this particular model is the long latency period required to develop invasive ductal adenocarcinoma [52,58]. To overcome the long latency period, researchers developed the LSL-KRAS^G12D^; LSL-Trp53^R172H^; PDX-1-Cre (KPC) mouse. The addition of a p53 mutation (R172H) accelerated pancreatic tumorigenesis and ameliorated the long latency period and infrequent tumor development with the LSL-KRAS^G12D^; PDX-1-Cre mouse model [58]. A logical progression in the development of GEMMs was to target other tumor suppressor genes known to be dysregulated in PDAC. For example, a conditional loss of the INK4a/ARF (CDKN2A) locus generated more rapid development of PanIN lesions, decreased tumor latency, a more undifferentiated histology, and the dissemination of metastatic lesions to the liver and lungs in the LSL-KRAS^G12D^ background [59,60]. A second example examined the loss of the tumor suppressor SMAD4 in the mutant LSL-KRAS^G12D^ background. Interestingly, the conditional loss yielded the formation of cystic type lesions that are seen in human disease [61,62,63]. Several comprehensive reviews have been published with more in-depth analysis of the multitude of GEMMs in PC that is beyond the scope of this review [64,65].

A major advantage to this type of GEM model is the ability to investigate and identify biomarkers for early disease detection and therapeutic intervention at relatively early disease stages. GEMMs present an opportunity to develop novel diagnostic methods to improve early detection. Toward this goal, Fendrich et al. (2011) used 18F-fluorodeoxyglucose, a radiolabeled glucose analog, and positron emission tomography (FDG-PET) imaging approaches to detect glucose metabolism in precursor lesions of pancreatic cancer [66]. This study determined that PanIN lesions as well as invasive pancreatic carcinoma had an increased signal in the pancreatic region, while no signal was detected in wild type mice [66]. Further, Faca et al. (2008) detected KRAS^G12D^ in the plasma of GEMMs of PDAC in which tumor initiation and progression were mediated by oncogenic KRAS^G12D^ and a conditional loss of the INK4a/Arf locus [67]. These investigators identified a panel of proteins that they are evaluating as potentially predictive of PDAC development [67]. GEM models of PC have been used to evaluate treatment regimens. These studies used non-invasive imaging techniques to assess efficacy and pharmacokinetic data and demonstrated that their data in GEM models were similar to data from human clinical trials [68]. GEM models may be useful predictors of chemotherapeutic response in human patients.

GEM models do, however, have several disadvantages. Development of these models is labor intensive and once established are costly to maintain [69]. While GEM models can develop tumors with 100% penetrance, time to tumor initiation and progression can be greater than 12 months. In currently available models that include expression of mutated KRAS, KRAS^G12D^ is not completely restricted to the pancreatic tissue [70,71]. Further, monitoring tumor progression requires specialized imaging equipment that is not available to many laboratories [72]. Finally, tumors are of murine origin and may not precisely recapitulate human PDAC. Despite these limitations, GEMMs have been used to generate useful and clinically relevant data and are important models for the study of PDAC.

## 3. Xenograft Models

Prior to the development of xenograft models, in vivo studies were performed using syngeneic or allograft mouse models. This in vivo model system was developed over 50 years ago and were used to study a variety of malignancies, such as colon cancer, breast cancer, and lung cancer [73,74,75]. Syngeneic murine models of PDAC were limited by the available murine cell lines to graft. This type of model has largely been replaced with the use of immunocompromised mice as tumor hosts. The development and characterization of immunodeficient mice can be regarded as a major milestone in oncology research. In 1966, the first BALB/c Nude (nu/nu) mouse was characterized [76]. A mutation in the *Foxn1* gene produced a mouse that lacked hair and a functional thymus, limiting the immune system to a low population of T-cells, minimal T-cell dependent response to antigens, and antibody response confined to the IgM class [76,77]. The impaired immune system of these mice allowed subcutaneously injected human cells to produce viable tumors in vivo. A variety of immunocompromised mouse strains are now available, and are compared in more detail in subsequent sections of this review.

Immunocompromised mouse strains made are now widely used as hosts for human tumors generated from essentially all human cell lines, termed cell line derived xenografts (CDX). These models reliably produce tumors within four to six weeks, depending on tumor cell inoculum, and provide a reproducible system amenable to statistical analysis [78]. PC CDX models include subcutaneous, orthotopic, and metastatic models [79,80,81]. These models have been used for drug screening, pharmacokinetic and pharmacodynamics assessments, and detecting toxicities [82,83,84]. However, models generated with cell lines lack the tumor cell and stromal cell heterogeneity of primary pancreatic tumors, and the ability to predict clinical response with this type of model is not always reliable. Tumors derived from pancreatic cancer cell lines lack heterogeneity due to multiple reasons [85]. These may include genetic adaptation to growth in vitro on plastic for many decades, which is not representative of primary tumors [85,86].

### 3.1. Generation of Patient-Derived Xenograft (PDX) Models

Patient-derived xenograft (PDX) models address the limitations of CDX models. These models are derived by direct implantation of primary human tumor specimens into immunocompromised mice. The rationale for the generation of such models is the hypothesis that these models would more closely resemble human disease and will more accurately predict response to given therapeutic regimens [87,88,89,90]. Direct implantation of tumor specimens circumvents time in vitro during which genetic selection or clonal expansion might occur. Human stromal elements are implanted with tumor cells in host mice and studies conducted to examine the duration for which human stromal elements are retained in PDX models. For example, Delitto et al. showed human stromal elements were present in first generation of PDAC PDX models but did not expand with tumor growth and were replaced with murine stroma [91]. Similar results have been observed in colorectal cancer PDX models [92,93].

Subcutaneous tumors require a minimally invasive surgical procedure and tumor growth is easily monitored [94,95]. Orthotopic tumors may more closely resemble primary human tumors and are more likely to produce metastatic lesions than subcutaneous tumors, but monitoring tumor progression is more difficult [72,96,97]. Methods to generate subcutaneous and orthotopic pancreatic PDXs have been detailed in several publications [94,98]. Once successful growth is established in mice tumor tissue can be serially transplanted into subsequent cohorts of mice, to provide adequate numbers of a given tumor for multiple applications and statistical analysis. Tumor tissue can be cryopreserved and repositories maintained. Undoubtedly, molecular and/or genetic ‘drift’ will occur in later passages or generations; however, *KRAS* mutational status in PDAC PDX tumors has been documented to be maintained for up to 39 generations when propagated in vivo [99].

PDAC PDXs develop ~1–4 months after implantation of a primary tumor specimen, with a “take rate” or successful growth of tumors reported to be 20–80% [44]. Successful engraftment depends predominantly on the number of viable tumor cells in the implanted specimen, the ratio of tumor cells to stroma, and the presence or absence of necrotic areas in the specimen [44]. In our hands, implantation of primary tumor specimens within an hour of resection produced an 85% take rate [100]. In addition, we observed a statistically significant correlation of take rate and size of the primary tumor from which the specimen was obtained. Specimens from tumors that were at least 2.5 cm in one dimension had higher engraftment rates than smaller tumors [100]. Consistent with this observation, other laboratories have reported that failure to engraft correlates with a more favorable clinical prognosis [101,102].

### 3.2. Mouse Strains for Generation of PDX Models

Another factor that impacts successful tumor engraftment is the strain of immunodeficient mouse chosen as host. Following the identification of the athymic nude mouse strain, a variety of immunocompromised strains have been identified and bred for research purposes. The mouse strains most commonly used to generate PDX models include the severe combined immunodeficient (SCID) strain, the non-obese diabetic (NOD-SCID) hybrid mouse strain, and the NOD-SCID Interleukin-2 receptor gamma (γ) chain deletion (NOD-SCID IL2rg^−/−^; NSG) strain [103,104,105]. These models differ in their residual immune components. SCID mice were first reported in 1983, following the observation of spontaneous agammaglobulinemia in CB-17 mice [103]. In these mice, a mutation in the DNA-activated, catalytic subunit of the protein kinase (*Prkdc*) gene yielded a severe combined immunodeficiency disease (*Prkdc^scid^*): these mice lack functional T- and B-cells [106,107]. This DNA-dependent protein kinase (*Prkdc*) is essential for joining non-homologous ends of DNA. Dysfunctional *Prkdc* impairs T- and B-cell function. These mice retain functional natural killer (NK) cells [108]. Hybrid NOD-SCID mice have fewer NK cells than SCID mice, and the take rate after tumor specimen implantation is higher than in SCID mice mainly due to the increased immunodeficiency [109]. A third model is a hybrid of NOD-SCID mice and IL2rg^−/−^ (NSG) mice. The IL-2R γ-chain contributes to Interleukin receptor signaling [110,111]. Deletion of the DNA locus encoding IL-2Rγ impairs T- and B-cell development and function, and prevents NK cell development [112,113,114]. All three models are suitable for development of pancreatic PDX models.

### 3.3. Comparison of Pancreatic PDX Tumors with Tumors of Origin

The utility of preclinical models depends in large part on how faithfully they recapitulate human disease. Several laboratories have documented that PDAC PDX models retain specific morphologic, genetic, histologic, and differentiation characteristics of the tumors from which they were derived, and human stromal components have been observed associated with subcutaneous PDX tumors for the first generation in vivo [89,115]. When a tumor specimen is implanted, human stromal elements are implanted as a component of the tumor specimen. As tumors are passaged in vivo, human stroma is replaced by murine stroma. The microenvironment of early passage PDX tumors comprises human tumor cells + human stroma, while subsequent passages are comprised of human tumor cells + predominantly murine stroma. Further, human tumor cells have been detected in the blood of the mice bearing pancreatic PDX tumors, suggesting that tumors retain metastatic potential [116]. Retention of primary tumor morphology and differentiation status is consistent with PDX models of other tumor types such as colon and breast cancer [117,118].

In addition to histological similarities, several laboratories report fidelity of multiple characteristics common to primary PDAC tumors [99,119]. Initial studies focused on sequencing DNA loci encoding driver genes specific to PDAC and immunohistochemistry (IHC) to determine concordance in expression of specific gene products in primary and PDAC PDX tumors. For example, Rubio-Viqueira et al. determined that *KRAS* mutational status of the primary tumor was retained and stable for at least three passages in 12 of 12 models [120]. The study compared first, second and third generation PDXs with primary tumors of origin. Of the 12 primary tumors, 10 harbored specific, single copy *KRAS* mutations (KRAS WT/MUT) that were maintained for the duration of the study. The remaining two primary tumors also harbored a single copy *KRAS* mutation: first and second generation tumors harbored the same single copy mutation, and the third generation tumors were homozygous for this mutation. In all models, *KRAS* mutation was maintained. The authors also used IHC to demonstrate that SMAD4 expression levels in 10 of 12 models were similar to those of the tumor of origin [120]. Several labs have looked more in depth into the genomic stability of pancreatic PDX models. Mattie et al. used copy number variation analysis, gene expression analysis, miRNA microarrays and mutation analysis to document a high degree of similarity between lower (5th generation) and higher passaged (>30 generations) PDX tumors and tumors of origin [99]. Additionally, Jung et al. (2016) performed exome sequencing on 8 pairs of pancreatic primary tumors and corresponding PDX models, utilizing a panel of 409 cancer-related genes to determine genomic conservation [119]. Clustering analysis of the exome sequencing data showed a high degree of concordance (range 90.2–97%) among the primary tumor and PDX model.

### 3.4. Biomarker Identification Using Pancreatic PDX Models

Diagnosis of PC remains challenging in the clinic, as patients often present with non-specific symptoms and there are no early screening methods for PC. Identification of tumor biomarkers to detect PDAC, have the potential to aid diagnosis, improve prognosis, and monitor response to treatment. While validated biomarkers have not been identified for PC, PDX models with high genetic and molecular fidelity to tumors of origin have been used to identify potential biomarkers for this tumor type. This area of investigation is described below and summarized in Table 1.

The first proof of concept study to address whether pancreatic PDX models could be used to identify markers of response to treatment was conducted in 2006 by the Hidalgo group [120]. A panel of 14 pancreatic PDXs was established in nude mice. This group of investigators observed that relatively high levels of expression of deoxycytidine kinase (dCK), an enzyme responsible for gemcitabine activation, were associated with sensitivity to gemcitabine. This observation suggested that levels of dCK expression might be used as a biomarker for response to gemcitabine. Further, the authors demonstrated the feasibility of testing multiple drugs for efficacy in all 14 PDX models to simulate a phase II clinical trial [63].

A second study by the Hidalgo group investigated factors influencing engraftment rates and predictability of clinical outcome in PDX models derived from surgically resectable patients with PDAC [121]. A total of 94 patients underwent surgical resection and 69 chemotherapy naïve tumors were implanted into nude mice and 42 were successfully engrafted. The authors found that loss of SMAD4 expression and successful engraftment was a significant predictor of shorter survival in patients (*p* = 0.006). Gene set enrichment analysis (GSEA) performed on microarray data of patient tumors showed gemcitabine resistance was associated with an upregulation of the stroma and stem cell pathways, specifically Notch signaling. A key feature of this study was as a clinical trial, relevant clinical parameters could be collected from patients and used to validate PDX model results.

In 2014, Torphy et al. used pancreatic PDX models to determine if levels of circulating tumor cells (CTCs) comprised biomarkers of response to the oral phosphatidylinositol-3 kinase inhibitor BKM120 [116]. Cohorts of mice received either vehicle or BKM120 for 28 days and CTCs were detected in peripheral blood smears using an immunofluorescence assay with antibodies to human cytokeratin 8/19 on days 0 and 28 of treatment. The data showed a decrease in the median number of CTCs in blood, from 26.6 on day 0 to 2.2 on day 28. The authors proposed that detection of CTCs in blood samples could be used as a proxy for response to chemotherapy in patients with PDAC.

As a fourth example, Dutta et al. used pancreatic PDX models to develop a novel non-invasive image-based biomarker to determine pancreatic tumor invasiveness or aggressiveness that could be utilized in the clinic [122]. Magnetic resonance microscopy was used to follow the conversion of radiolabeled pyruvate to lactate in vivo, and the data indicated that higher conversion rates were found in the faster growing PDX models. This finding was corroborated by immunohistochemistry (IHC) of lactate dehydrogenase (LDH) which is known to be expressed at relatively high levels in aggressive PDX tumor models [122]. As pancreatic cancer is difficult to detect early this could be a potential non-invasive way to determine the prognosis of the patient, as well as, monitor treatment in patients diagnosed with PDAC.

These studies demonstrate the utility of PDX models for identifying biomarkers of disease progression or response to therapy. A principle advantage of this type of model system is exemplified in the Hidalgo clinical trial [121]. Because PDX models retain genomic and molecular characteristics of the tumors from which they were derived, prospective studies for biomarkers can be conducted and preclinical data compared with clinical outcome.

### 3.5. The Use of Pancreatic PDX Models for Preclinical Drug Evaluation

No approved chemotherapeutic regimens produce durable clinical responses in patients with PDAC. This section discusses five studies that used PDAC PDX models to demonstrate the potential utility of novel single or combination regimens for treating this tumor type. The discussion is summarized in Table 2.

The first of these studies focused on an approach to circumvent or overcome resistance to gemcitabine. Gemcitabine is a frontline agent for treating PDAC patients, and has some efficacy in the clinic but is not curative as a single agent or in any combination evaluated thus far. Fine needle aspirates were taken from 11 pancreatic PDX models with varying levels of gemcitabine sensitivity and grown ex vivo with or without 6 hour exposure to gemcitabine. [123]. Fine needle aspirates were subjected to low density microarray analysis of RNA extracted from with or without gemcitabine identified polo-like kinase 1 (PLK1) as a gene of therapeutic interest. Comparison of the therapeutic efficacy of gemcitabine alone or in combination with the PLK1 inhibitor rigosertib (ON01910.NA) in mice bearing a panel of three PDAC PDX tumors with intermediate resistance to gemcitabine (*n* = 1) or gemcitabine refractory tumors (*n* = 2). In the gemcitabine refractory tumors, the combination treatment induced tumor regressions and suggested rigosertib could be a potential sensitizing agent in a subset of gemcitabine resistant tumors. This study was one of the first designed to target a specific gene product to overcome gemcitabine resistance in PDX models of PDAC.

A second study used PDX models of PDAC to investigate potential efficacy of polo-like kinase 4 (PLK4) [124]. PLK4 plays a role as a mitotic kinase that functions late in the cell cycle [132,133]. CFI-400945 was tested against a panel of six individual orthotopic pancreatic PDX models with a variety of phenotype such as growth characteristics, genetic abnormalities, and levels of hypoxia. A reduction in tumor growth was observed in four of six models when treated with this single agent PLK4 inhibitor (*p* < 0.05).

A third preclinical study used PDAC PDX models to address whether the Chk1 inhibitor AZD7762 could sensitize tumor cells to gemcitabine [125]. The data showed that gemcitabine or AZD7762 alone or in combination suppressed tumor growth in two subcutaneous pancreatic PDX models. Interestingly, the combination gemcitabine and AZD7762 depleted the cancer stem cell (CSC) population as measured by FACS analysis. Furthermore, secondary tumor formation assays were performed on each treatment group and monitored for 10 weeks for tumor initiation in vivo. The authors observed that 83% of mice treated with gemcitabine alone developed tumors, while only 43% of mice treated with the combination developed tumors (*p* < 0.05).

Our lab has also used PDAC PDX models to evaluate the efficacy of inhibitors of the bromodomain and extraterminal (BET) family of proteins in treating this tumor type [126,127]. BET family members (BRD2, BRD3, BRD4, and BRDT) recognize acetylated lysine residues on histones and recruit proteins required for transcription of specific genes [134,135,136]. The small molecule BET bromodomain inhibitor JQ1 competitively binds to bromodomain motifs on BET proteins to disrupt gene transcription. We reported that JQ1 suppressed tumor growth in five independent PDX models of PDAC and that JQ1 decreased the expression of CDC25B, a G2/M cell cycle regulator, in 4 of 5 JQ1-sensitive PDX models using NanoString platform [126]. In a follow up study, we observed that JQ1 decreased expression of DNA repair proteins RAD51 and Ku80, and simultaneously increased levels of the DNA damage marker γH2AX in PDX models [127]. These data suggested the hypothesis that decreased DNA repair by JQ1 would sensitize pancreatic tumor cells to PARP inhibitors. Efficacy studies using two PDAC PDX models demonstrated that the BET inhibitor JQ1 + the PARP inhibitor olaparib was well tolerated and was more effective than either drug alone [127]. The combination of BET inhibitor and the PARP inhibitor in PDAC warrants further investigation.

### 3.6. Pancreatic PDX Models in Precision Medicine and Clinical Relevance

PDX models clearly have utility in preclinical studies of pancreatic cancer. However, the degree to which these models will be useful in designing therapeutic regimens for specific patients or tumor types is unknown. Pioneering work in this area by Hidalgo and colleagues provides reason for optimism. Below we summarize studies that directly address the likelihood that PDAC PDX models comprise useful tools for designing therapeutic regimens.

Two published studies associated with a clinical trial conducted by Hidalgo et al. demonstrate that efficacy data from PDAC PDX experiments have relevant clinical application [128,129]. The first study was a pilot study that enrolled 14 patients with advanced solid tumors, including four patients with PDAC [128]. PDX models were established from resected tumors (*n* = 6) or metastatic lesions (*n* = 8) and the anti-tumor efficacy of 63 compounds, in 232 different combinations was evaluated in these 14 PDX models. One patient died prior to obtaining experimental data. The remaining 13 patients were treated with the combination predicted by PDX data to be the most effective. There were 11 patients who received 17 prospectively guided treatments based on PDX data, and 15 of those treatment regimens resulted in durable partial remissions. Specific to the PDAC patients, 15 unique compounds were investigated as single agents, as well as, 7 combination treatments to determine drug efficacy.

One of the patients in this clinical trial was known to have gemcitabine resistant PDAC [129]. PDX data predicted that this tumor would be sensitive to the DNA damaging agents mitomycin c and cisplatin. The patient was treated with mitomycin c as a single agent, and achieved a partial response of 22 months’ duration before disease progression was observed. The patient was again treated with mitomycin c, but renal toxicity mandated a change to cisplatin. The patient responded to cisplatin and remained symptom free for three years. The demonstrated correlation between PDX data and clinical response was unprecedented.

More recent advancements include efforts to develop integrative platforms to guide patient therapy, such as the comprehensive approach reported by Gao et al. These investigators used 277 PDX models (*n* = 42 PDAC PDXs) of breast, colon, lung, gastric, and pancreatic cancers to evaluate 62 unique treatments to mimic a phase I/II clinical trial setting [130]. The study was done with one mouse per model per treatment with a goal of identifying association between drug response and specific genomic characteristics for better prediction of patient response in clinical trials. In a proof of concept study specific to PDAC, Witkiewicz et al. used both patient-derived cell lines and PDX models to identify potential chemotherapeutic interventions in PDAC [131]. In one patient, high-throughput screening of primary tumor material identified a sensitivity to the MEK inhibitor AZD6244, and PDX model derived from the same patient showed a similar sensitivity (*p* < 0.01). The investigators of this study suggest this type of approach may be valuable for identifying useful drug targets and for validating these targets.

### 3.7. Limitations of Pancreatic PDX Models

Despite notable utility, pancreatic PDX models have limitations. First, these models are labor intensive to establish, costly to maintain, and may have a significant lag between engraftment and first passage. Tissues for engraftment are limited, as only 20% of the ~56,000 patients diagnosed this year will be eligible for surgical resection, and tumor take rates vary between 20% and 85% among laboratories [44,100]. The feasibility of generating PDX models with fine-needle aspirate biopsies could increase the potential pool of patient samples if implemented [129]. Second, PDX models, as well as all other models, is the loss of human stroma in early passages. Human stromal tissue is replaced with murine stroma, usually within the first generation [91,92,93], and the relevance of human PDAC tumor-stroma interaction must be addressed in individual studies. However, recent advances in transcriptomic analysis has allowed for such tumor-stroma interaction analysis in silico. For example, Moffitt et al. utilized computational methods to define tumor, stroma, and normal tissue genetic signatures in PDAC. The study identified and characterized two tumor subtypes (basal-like and classical), as well as normal or activated stroma [137]. In PDAC PDX models, both tumor signatures aligned with known human transcripts, while the murine transcripts associated with the stromal signatures [137]. An additional study used multiomics and computational methods to analyze tumor and stromal gene signatures to identify novel drug targets in PDAC [138]. In this study, the cholesterol transporter Niemann-Pick C1-Like 1 Protein (NPCL1L) was determined to be increased in PDAC cells. NPCL1L was evaluated as a potential drug target by treating mice bearing two PDAC PDX models to the cholesterol absorption inhibitor ezetimibe. Ezetimibe as a single agent inhibited tumor growth (*p* < 0.001).

Third, recent literature documents some genomic alterations in PDX models after serial passaging in mice. Ben-David et al. assessed the changes in the copy number alteration (CNA) landscape among 1,110 PDX samples across 24 tumor types including PDAC models [139]. This study observed CNAs in PDX models of all tumor types during the engraftment and early passage. These CNAs resulted primarily from expansion of pre-existing subclones within tumors, and not de novo events. These investigators proposed that these genetic differences between PDX tumors and their primary tumors of origin could alter therapeutic response and should be considered when interpreting drug efficacy and biomarker studies [139].

Fourth, PDX tumors are propagated in immunocompromised mice. It is well accepted that components of tumor, stroma, and the immune system interact in the tumor microenvironment [140,141,142]. In PDX models, a majority of stromal tissue and residual immune competent cells, if any, are of murine origin; therefore, these models cannot be regarded as having a true tumor microenvironment. The use of PDX models to evaluate agents that target tumor-stroma interaction requires careful interpretation, and have limited use for evaluating agents that regulate immune-mediated anti-tumor efficacy. To overcome this problem, several laboratories have begun to develop “humanized mice” PDX models, in which components of the human immune system are expressed into mice [143,144]. One such strategy is the transplantation of CD34^+^ hematopoietic stems cells (HSCs) isolated from human umbilical cord blood, bone marrow, or peripheral blood into irradiated NSG mice [145,146]. Engraftment is considered successful when human CD45+ immune cells surpass 25% in peripheral blood, allowing for PDX implantation. Using this strategy, Morton et al. demonstrated in PDX models of head and neck cancer that human B- and T-cells migrated to tumor tissue and induced pro-lymphogenic factors [147]. With specific relevance to PDAC, Ames et al. used a humanized mouse model to demonstrate that human NK cells infiltrated orthotopic PDAC tumors, preferentially targeted the CSCs, and inhibited tumor growth [148]. This type of model, however, does have the potential for the development of graft versus host disease and may show impaired differentiation and maturation of immune cells due to species specific cytokines. Residual murine macrophages and granulocytes may also modify responses to human CD45+ cells. Transgenic approaches are being used to further reduce host immune function and mitigate these limitations. Although the use of humanized mouse models with PDAC PDX tumors is in its nascent stages, we anticipate their increased use in the future.

## 4. Conclusions

The 5-year survival for patients with nonresectable pancreatic cancer is ~9% [1]. Each type of preclinical model used to characterize the genotype and phenotype of pancreatic tumors—2D immortalized cell lines, 3D organoids, GEMMs, and PDX models—has advantages and disadvantages, and the model of choice will depend on the experimental goal. However, the degree to which PDAC PDX models have been shown to retain characteristics of their tumors of origin and their demonstrated utility in reflecting clinical response are viewed as distinct advantages for identifying markers for the early detection of PDAC, the characterization of molecular and genetic changes critical to PDAC tumor progression, and the evaluation of therapeutic efficacy of treatment regimens.

## Figures and Tables

**Table 1:** Published studies discussed in this review that describe genetic and molecular characteristics of PDAC PDX models and biomarker identification efforts.

Study	Type of Study	Focus of Study	Observation	Reference
Mattie et al. (2013)	Model validation	Expression profiling	High correlation of gene expression profiles between early and late passage PDAC PDX tumors.	[99]
Rubio-Viqueira et al. (2006)	Model validation	Genetic stability, expression profiling	KRAS status and SMAD4 expression level conserved in 10-12 PDAC PDXs, compared to tumors of origin.	[120]
	Biomarker	Gemcitabine sensitivity	Higher level dCK expression predicted greater response to gemcitabine.	
Jung et al. (2016)	Model validation	Genetic stability	>90% sequence similarity between primary tumor and PDAC PDX models.	[119]
Garrido-Laguna et al. (2011)	Biomarker	Gemcitabine sensitivity	Gene enrichment analysis showed increases in expression of genes that contribute to Notch signaling and to the production of stroma in gemcitabine resistant tumors.	[121]
Torphy et al. (2014)	Biomarker	Circulating tumor cells	Treatment with BKM120 decreased the number of circulating tumor cells.	[116]
Dutta et al. (2019)	Biomarker	Glucose metabolism	Increased conversion of radiolabeled pyruvate to lactate in PDAC PDXs with relatively rapid rates of proliferation.	[122]

**Table 2:** Preclinical studies discussed in this review that evaluate drug efficacy in PDX models of PDAC.

Study	Study Type, Preclinical	Number of Models Used	Tumor Location	Drug Target(s)	Drug (s) Evaluated	Reference
Jimeno et al. (2010)	Efficacy	3	Subcutaneous	PLK1	rigosertib, gemcitabine	[123]
Lohse et al. (2017)	Efficacy	6	Orthotopic	PLK4	CFI-400945	[124]
Venkatesha et al. (2012)	Efficacy	2	Subcutaneous	CHK1	AZD7762	[125]
Garcia et al. (2016)	Efficacy	5	Subcutaneous	BET proteins	JQ1	[126]
Miller et al. (2019)	Efficacy	2	Subcutaneous	BET proteins, PARP	JQ1, olaparib	[127]
Hidalgo et al. (2011)	Precision medicine	4	Subcutaneous	Multiple	15 agents, 15 monotherapy 7 combinations	[128]
Villarroel et al. (2011)	Precision medicine	1	Subcutaneous	DNA synthesis DNA replication	mitomycin C, cisplatin	[129]
Gao et al. (2015)	Precision medicine	42	Subcutaneous	Multiple	38 agents, 36 monotherapy 26 combinations	[130]
Witkiewicz et al. (2016)	Precision medicine	2	Subcutaneous	MEK	AZD6244	[131]

## Abbreviations

CDX	Cell line derived xenograft
GEMM	Genetically engineered mouse model
IL2rg	Interleukin-2 receptor gamma chain
NOD	Non-obese diabetic
PC	Pancreatic Cancer
PDAC	Pancreatic ductal adenocarcinoma
PDX	Patient-derived xenograft
PanIN	Pancreatic intraepithelial neoplasm
SCID	Severe combined immune deficiency
